# Concordance of care processes between medical records and patient self-administered questionnaires

**DOI:** 10.1186/s12875-019-0979-7

**Published:** 2019-07-03

**Authors:** Cynthia Khanji, Mireille E. Schnitzer, Céline Bareil, Sylvie Perreault, Lyne Lalonde

**Affiliations:** 1 Faculty of pharmacy, University of Montreal, 2940 Polytechnique Road, Montreal, Quebec H3T1J4 Canada; 2 HEC Montréal, University of Montreal, 3000 Côte-Sainte-Catherine Road, Montreal, Quebec H3T2A7 Canada; 3 Sanofi Aventis Endowment Chair in Drug Utilization, Montreal, Canada; 4 Sanofi Aventis Endowment Chair in Ambulatory Pharmaceutical Care, Montreal, Canada

**Keywords:** Primary care, Quality indicators, Data quality, Documentation, Medical record, Patient self-administered questionnaire

## Abstract

**Background:**

Despite the increasing use of medical records to measure quality of care, studies have shown that their validity is suboptimal. The objective of this study is to assess the concordance of cardiovascular care processes evaluated through medical record review and patient self-administered questionnaires (SAQs) using ten quality indicators (TRANSIT indicators). These indicators were developed as part of a participatory research program (TRANSIT study) dedicated to TRANSforming InTerprofessional clinical practices to improve cardiovascular disease (CVD) prevention in primary care.

**Methods:**

For every patient participating in the TRANSIT study, the compliance to each indicator (individual scores) as well as the mean compliance to all indicators of a category (subscale scores) and to the complete set of ten indicators (overall scale score) were established. Concordance between results obtained using medical records and patient SAQs was assessed by prevalence-adjusted bias-adjusted kappa (PABAK) coefficients as well as intraclass correlation coefficients (ICCs) and 95% confidence intervals (95% CI). Generalized linear mixed models (GLMM) were used to identify patients’ sociodemographic and clinical characteristics associated with agreement between the two data sources.

**Results:**

The TRANSIT study was conducted in a primary care setting among patients (*n* = 759) with multimorbidity, at moderate (16%) and high risk (83%) of cardiovascular diseases. Quality of care, as measured by the TRANSIT indicators, varied substantially between medical records and patient SAQ. Concordance between the two data sources, as measured by ICCs (95% CI), was poor for the subscale (0.18 [0.08–0.27] to 0.46 [0.40–0.52]) and overall (0.46 [0.40–0.53]) compliance scale scores. GLMM showed that agreement was not affected by patients’ characteristics.

**Conclusions:**

In quality improvement strategies, researchers must acknowledge that care processes may not be consistently recorded in medical records. They must also be aware that the evaluation of the quality of care may vary depending on the source of information, the clinician responsible of documenting the interventions, and the domain of care.

**Electronic supplementary material:**

The online version of this article (10.1186/s12875-019-0979-7) contains supplementary material, which is available to authorized users.

## Introduction

In recent years, measuring and improving quality of care has been a thriving area of interest both in North America and internationally. High-quality data which are a result of good documentation practices are essential not only for patient care but for monitoring quality of care and implementing quality improvement strategies. Despite the increasing use of medical records to measure quality of care, studies have shown that the validity of medical records is suboptimal [1–3].

Medical records are a key source of information about care processes (e.g. diagnosis, treatment, referral, prescribing) and clinical outcomes (e.g. morbidity, mortality, health-related quality of life) [4, 5]. However, their accuracy may be limited by time pressures and delayed recording which can lead to poor documentation [1, 3, 4, 6]. Other potential drawbacks are the lack of documentation standards [7, 8] and the fact that some of the data recorded in medical records depend on patient self-reported information such as symptoms and lifestyle behaviors [1, 3]. Self-reported data sources such as patient self-administered questionnaires (SAQs) have some advantages over medical records. They are developed in line with research objectives and data generated by SAQs provide insight into the patient’s point of view [9]. However, patient SAQs are dependent on the amount of information the patient remembers, considers relevant, and is willing to share [10, 11].

Several studies have assessed concordance between information collected through SAQs and medical records, but have focused on diagnoses [12], health services utilization [13], medication [14], and specific symptoms [15] or diseases [7]. In a primary care setting, particularly in the context of an intervention program aimed at improving quality of care, little is known on the concordance of care processes evaluated using medical records and patient SAQs. As part of a participatory research program (TRANSIT study) dedicated to TRANSforming InTerprofessional clinical practices to improve cardiovascular disease (CVD) prevention in primary care, members of the primary care community developed a set of 81 process indicators (TRANSIT indicators). Among those, 10 indicators were evaluated through both medical record review and patient SAQs. The objectives of this study are to assess the concordance of care processes evaluated through medical record review and patient SAQs using the TRANSIT quality indicators and identify patients’ sociodemographic and clinical characteristics associated with agreement between the two data sources.

## Methods

### Study design

The TRANSIT study is a three-phase participatory research program aimed at supporting continuous improvement of CVD prevention in primary care. Details on the study design of each phase are described elsewhere (Phase I [16], Phase II [17], and Phase III [18]). The current study is a secondary analysis of the data collected in the cluster randomized controlled trial (Phase III) and has been approved by the research ethics board of the *Centre de santé et de services sociaux de Laval* (CSSSL) (2013–2014/04–02). Every participating clinician and patient signed an informed consent form.

### Study population

Family medicine groups (FMGs) were recruited in the large urban area of Laval, Quebec, Canada. To be eligible, each FMG had to build an interprofessional facilitation team (IFT) composed of voluntary participating clinicians. The role of the IFT was to promote the implementation of the program among colleagues inside and outside the FMG. It was required that each IFT include at least one nurse, two physicians, and one community pharmacist, as well as one nutritionist, kinesiologist, or psychologist. Some FMGs invited their administrative staff to join the IFT, but this remained optional. Pharmacists, nutritionists, kinesiologists, and psychologists could be recruited from outside the FMG. Each FMG was asked to recruit 100 participating patients. To be eligible patients had to meet the following criteria: 1) have 18 years old and older; 2) be at moderate or high risk of CVD as evaluated by the Framingham CVD risk score [19]; 3) have hypertension, dyslipidemia or diabetes that was either uncontrolled or for which pharmacotherapy had initiated in the past 12 months; and 4) have at least two other chronic diseases, excluding CVD and CVD risk factors. Patients were excluded if they did not meet the 4 inclusion criteria or if they received home care services.

A total of eight FMGs, 98 clinicians and 759 patients participated in Phase III of the TRANSIT study. Six FMGs including 590 patients were randomized into the “facilitation” group while two FMGs including 169 patients were randomized into the “passive diffusion” group. Four FMGs used paper medical records while the other four used paper and electronic medical records (EMRs).

### Quality indicators

The TRANSIT indicators were developed through a rigorous participatory process involving researchers, health care managers, clinicians, patients, and family members (see Additional file 1). The full set includes 81 process indicators (see Additional file 2: Table S1) that are evaluated using medical records (*n* = 60), pharmacy renewal charts (*n* = 4), and patient SAQs (*n* = 17). Of the 17 indicators documented through patient SAQs, ten were also documented through medical record review. These indicators read as follows: 1) instructions for home blood pressure monitoring (indicator [IND] 29,30); 2) instructions for home glucose monitoring (IND49,50); 3) education for self-management education for diabetes (IND58,59); 4) referral to a clinician other than a physician and a nurse (IND66,67); 5) referral to a group class offered by the local care center (IND70,71); 6) referral to a community resource (IND73,74); 7) discussion on lifestyle habits during a meeting with the nurse (IND79,80); 8) discussion of the impact of chronic health conditions on the patient’s quality of life (IND82,83); 9) establishment of personal targets for lifestyle changes (IND84,86); and 10) use of the TRANSIT health booklet (IND88,89). The ten pairs of indicators were used in this study to evaluate the concordance of care processes between medical records and patient SAQs. Reliability of the indicators documented through medical record review (IND30, IND50, IND59, IND66, IND70, IND73, IND79, IND82, IND84, and IND89) was previously assessed in a psychometric analysis [20, 21]. Test–retest reliability was almost perfect while inter-rater reliably was substantial to almost perfect [22].

### Data collection

Five research assistants attended a two-week training session described in the Additional file 1. Once the training was completed, the research assistants documented the TRANSIT indicators retrospectively over the 14 months prior to the end of the TRANSIT study. For each patient, they assessed whether the care processes were in compliance (yes/no) with the indicators or were not applicable.

A patient SAQ was mailed to patients at baseline and at the end of the study (t_14_) with a postage-paid envelope for returning the completed version of the questionnaire. Clear instructions on how to complete the patient SAQ were found on the first page of the questionnaire. Patients could communicate with a research assistant by phone if they had any questions concerning the questionnaire. The patient SAQ sent at baseline (*n* = 101 items) was used to assess quality indicators and collect patient characteristics while the one sent at t_14_ (*n* = 97 items) was used to collect data on quality indicators. Items included in the questionnaires were mainly close-ended multiple choice and short open-ended questions.

### Statistical analyses

Baseline characteristics of participants were described using means and standard deviations for continuous variables as well as numbers and proportions for categorical variables. At t_14_, the compliance (yes/no) to each indicator (individual compliance scores) as well as the mean compliance (number of compliant indicators/number of applicable indicators) to all indicators of a category (subscale compliance scores) and to the complete set of indicators (overall compliance scale score) were established.

The concordance of care processes was determined by assessing correlations between results obtained using medical records and those obtained using patient SAQs. Patients with missing data for care processes in either data source during the 14 months preceding t_14_ were not included in the respective statistical analyses. Percentage of concordant evaluations and prevalence-adjusted bias-adjusted kappa (PABAK) coefficients [23] were computed for each pair of indicators while intraclass correlation coefficients (ICCs) and 95% confidence intervals (CI) were computed for subscale and overall compliance scale scores. PABAK coefficients were used because, unlike kappa coefficients, they are not influenced by prevalence and bias between raters [23]. PABAK values less than 0, between 0.00 and 0.20, between 0.21 and 0.40, between 0.41 and 0.60, between 0.61 and 0.80, and greater than 0.81 indicate poor, slight, fair, moderate, substantial, and almost perfect agreement, respectively [22]. ICCs values less than 0.5, between 0.5 and 0.75, between 0.75 and 0.9, and greater than 0.90 indicate poor, moderate, good, and excellent agreement, respectively [24].

To better understand if patient characteristics could influence self-report, an explanatory analysis was conducted. This analysis was conducted only among patients with compliant care processes in the medical record and no missing data in the patient SAQ. For each corresponding pair of indicators, generalized linear mixed models (GLMM) (binary logistic regression) with a random (cluster specific) intercept were used to identify patients’ sociodemographic and clinical characteristics associated with agreement of the indicator pair [25]. The sociodemographic characteristics included in this analysis were: age, sex, highest level of education completed, current work, and annual gross family income at baseline. The clinical characteristics included as covariates in the models were: intervention program group, CVD risk category estimated by the Framingham CVD risk score [19], smoking status, body mass index (BMI), diagnosis of hypertension, dyslipidemia, and diabetes as well as the achievement of therapeutic targets for hypertension, dyslipidemia, and diabetes at baseline. An odds ratio (OR) and 95% CI were computed for each pair of indicators. A two-sided alpha level of 0.05 was used to test statistical significance.

## Results

Of the 1024 eligible patients invited to participate to the TRANSIT study, 759 (74%) accepted. The patient SAQ response rate was 100% at baseline and 79% at t_14_. Among the ten indicators evaluated through both medical record review and patient SAQs, seven were applicable to all patients (*n* = 759 patients), one was applicable to hypertensive patients (*n* = 596), and two were applicable to diabetic patients (*n* = 508). Missing data varied from 3% (IND30: *n* = 15) to 5% (IND66, 70, 73, 79, 82, 84, 89: *n* = 36) in medical records and from 22% (IND82, 89: *n* = 166) to 33% (IND29: *n* = 194) in patient SAQs.

As reported in Table 1, baseline characteristics of the three sets of patients were similar in terms of age (all patients: 62 years; hypertensive patients: 63 years; diabetic patients: 62 years), sex (54%; 52%; 52%), and other sociodemographic and clinical characteristics. Compared to the total population, hypertensive and diabetic patients were less likely to have an annual gross family income higher than $50,000 (36%; 33%; 31%). Diabetic patients were also more likely to be at high risk of CVD (83%; 87%; 96%), to have uncontrolled hypertension (64%; 64%; 70%), and to have a BMI ≥ 30 kg/m^2^ (57%; 59%; 64%). However, they were less likely to have uncontrolled dyslipidemia (58%; 54%; 47%) than the total population.

**Table 1:** Baseline characteristics of the TRANSIT study’s participants

	All patients (*n* = 759)	Hypertensive patients (*n* = 596)	Diabetic patients (*n* = 508)
Sociodemographic characteristics^1^			
Age (years), mean (SD)	62 (11)	63 (11)	63 (11)
Males, n (%)	407 (54)	312 (52)	262 (52)
Highest level of education completed, n (%)			
None or elementary school	126 (17)	111 (19)	92 (18)
Secondary school	356 (47)	279 (47)	248 (49)
College technical school or university	268 (35)	199 (33)	161 (32)
Current work, n (%)			
Employed and/or self-employed	316 (42)	227 (38)	195 (38)
Unemployed, social security, and/or invalidity	49 (6)	36 (6)	39 (8)
Retired and/or stays home by choice	390 (51)	329 (55)	271 (53)
Annual gross family income, n (%)			
< $20,000	112 (15)	93 (16)	87 (17)
$20,000–$50,000	264 (35)	208 (35)	187 (37)
> $50,000	272 (36)	199 (33)	157 (31)
Clinical characteristics^1^			
CVD risk category, n (%)			
Moderate	123 (16)	76 (13)	20 (4)
High	633 (83)	519 (87)	487 (96)
Diabetes, n (%)	508 (67)	429 (72)	508 (100)
Uncontrolled diabetes^2^	403 (79)	342 (80)	403 (79)
Hypertension, n (%)	596 (79)	596 (100)	429 (84)
Uncontrolled hypertension^3^	380 (64)	380 (64)	301 (70)
Dyslipidemia, n (%)	717 (95)	575 (97)	486 (96)
Uncontrolled dyslipidemia^4^	418 (58)	312 (54)	228 (47)
Current smokers, n (%)	115 (15)	90 (15)	77 (15)
Body mass index, n (%)			
< 25 kg/m^2^	87 (11)	65 (11)	41 (8)
25–30 kg/m^2^	238 (31)	181 (30)	141 (28)
≥ 30 kg/m^2^	434 (57)	350 (59)	326 (64)

Abbreviations: CVD, cardiovascular disease; n, number; SD, standard deviation

^1^Data are missing if the total number of patients is different than 759 (all patients), 596 (hypertensive patients) or 508 (diabetic patients)

^2^Fasting blood glucose > 7.0 mmol/L, glycated hemoglobin > 7% and/or glucose 2 h post-prandial > 10.0 mmol/L

^3^Blood pressure ≥ 140/90 mmHg or ≥ 130/80 mmHg if diabetes and/or kidney disease

^4^Low-density lipoprotein cholesterol ≥2 mmol/L and/or apolipoprotein B ≥ 0.8gl/L

As reported in Table 2, in the “hypertension management” category, 50% of patients received instructions regarding blood pressure monitoring based on patient SAQs (IND29) compared to 15% based on medical records (IND30). In the “diabetes management” and the “interprofessional collaboration” categories, the percentages of patients reporting compliant care processes were also higher compared to the levels of compliance obtained using medical records. For the category “motivational interviewing and support for healthy lifestyle change”, levels of compliance based on patient SAQ and medical records were similar for the indicators “meeting with the nurse to discuss lifestyle habit” (77 and 78%) and “use of the TRANSIT health booklet” (41 and 46%). However, compared to medical records, compliance assessed by patient SAQ was lower for the indicator “impact of chronic health conditions” (18 and 34%) and higher for the indicator “personal lifestyle changes target” (76 and 55%). Overall, for seven out of ten indicators, the compliance as assessed by patient SAQ was higher than the compliance assessed by medical records. The percentage of concordant evaluations for each pair of indicators varied from 47 to 83%, with PABAK coefficients ranging from − 0.06 to 0.66.

**Table 2:** Concordance of individual compliance scores to TRANSIT indicators between data sources

Codes	Indicators	Patient SAQ	Medical record		
		Individual compliance scores n/N (%)	Individual compliance scores n/N (%)	Concordant observations n/N (%)	PABAK
Hypertension management					
IND29/30	Instructions for home BP monitoring	201/402 (50)	90/581 (15)	228/401 (57)	0.14
Diabetes management					
IND49/50	Instructions for home blood glucose monitoring	257/381 (67)	118/491 (24)	179/380 (47)	−0.06
IND58/59	Education for self-management education for diabetes	339/379 (89)	217/491 (44)	212/379 (56)	0.12
Interprofessional collaboration					
IND67/66	Referral to a clinician other than a physician and a nurse	358/583 (61)	339/723 (47)	431/575 (75)	0.50
IND71/70	Referral to a group class offered by the local care center^1^	234/573 (41)	129/723 (18)	383/565 (68)	0.36
IND74/73	Referral to a community resource^2^	99/562 (18)	26/723 (4)	446/554 (81)	0.61
Motivational interviewing and support for healthy lifestyle change					
IND80/79	Meeting with nurse to discuss lifestyle habits	452/586 (77)	562/723 (78)	479/578 (83)	0.66
IND83/82	Impact of chronic health conditions^3^ on the patient’s quality of life	105/593 (18)	245/723 (34)	394/585 (67)	0.35
IND86/84	Personal lifestyle changes target(s)	447/585 (76)	397/723 (55)	369/577 (64)	0.28
IND88/89	TRANSIT health booklet^4^ used	242/593 (41)	331/723 (46)	387/585 (66)	0.32

Abbreviations: BP, blood pressure; CI, confidence interval; n, number; N, total number; PABAK, prevalence-adjusted bias-adjusted kappa; SAQ, self-administrated questionnaire

^1^Group classes offered by the local care center include group class on diabetes, dyslipidemia, diabetes, healthy weight management, and smoking habits

^2^Community resources include resources for nutrition (organization) and physical activity (recreational center and walking club) as well as phone line for diabetes, depression, physiological help, physical activity, smoking cessation, and nutrition

^3^Chronic disease or risk factor

^4^The TRANSIT health booklet is personalized tool that allows the patient receiving care from multiple clinicians in various locations to share his/her medical information. It also helps the patient keep track of his/her progress

As reported in Table 3, subscale compliance scores (95% CI) obtained using patient SAQs varied from 41% (38–44) to 78% (74–81) while subscale compliance scores obtained using medical records varied from 15% (12–18) to 53% (50–56). Overall compliance scale scores obtained using patient SAQs and medical records were equal to 51% (49–54) and 37% (35–39), respectively. Concordance between patient SAQs and medical records, as measured by ICCs (95% CI), varied from 0.18 (0.08–0.27) to 0.46 (0.40–0.52) for subscale compliance scores and was equal to 0.46 (0.40–0.53) for the overall compliance scale score.

**Table 3:** Concordance of subscale and overall compliance scale scores to TRANSIT indicators between data sources

	Patient SAQ	Medical records	
	Compliance scores Mean (95% CI)	Compliance scores Mean (95% CI)	ICC (95% CI)
Subscale compliance scores for each category			
Hypertension management	50% (45–55)	15% (12–18)	0.18 (0.08–0.27)
Diabetes management	78% (74–81)	34% (32–36)	0.21 (0.12–0.31)
Interprofessional collaboration	41% (38–44)	23% (21–24)	0.39 (0.32–0.46)
Motivational interviewing and support for healthy lifestyle change	53% (51–54)	53% (50–56)	0.46 (0.40–0.52)
Overall compliance scale score	51% (49–54)	37% (35–39)	0.46 (0.40–0.53)

Abbreviations: CI, confidence interval; ICC, intra-class correlation coefficient; SAQ, self-administered questionnaire

As reported in Table 4, to examine the association between patients’ characteristics and the reporting of compliant care processes by the patient among those with compliant care processes based on the medical record, GLMM were used. Indicators related to hypertension management (IND29,30), diabetes management (IND49,50 and IND58,59), and interprofessional collaboration (IND66,67; IND70,71; and IND73,74) were not included in the explanatory analysis due to the large number of missing data and the high prevalence of noncompliant care processes in the medical record. For the four indicators related to motivational interviewing and support for healthy lifestyle change, no sociodemographic characteristics were significantly associated (increased odds: OR > 1 or decreased odds: OR < 1) with agreement between medical records and patient SAQ. In terms of clinical characteristics, smoking (OR = 0.38 [0.18–0.87]) was associated with decreased odds of agreement for the indicator related to a meeting with the nurse to discuss lifestyle habits (IND79,80) while a diagnosis of hypertension (OR = 3.36 [1.12–10.04]) was associated with increased odds of agreement for the indicators related to a personal lifestyle change target (IND84,86).

**Table 4:** Generalized linear mixed models to identify patients’ characteristics associated with concordance between data sources

	Meeting with the nurse to discuss lifestyle habits (*n* = 400 patients)			Impact of chronic health conditions on the patient’s QoL (*n* = 177 patients)			Personal lifestyle change target(s) (*n* = 281 patients)			TRANSIT health booklet used (*n* = 246 patients)		
	OR	95% CI	*p*-value	OR	95% CI	p-value	OR	95% CI	p-value	OR	95% CI	p-value
Sociodemographic characteristics												
Age	0.96	0.91–1.00	0.06	0.99	0.94–1.05	0.76	0.99	0.94–1.05	0.83	0.98	0.94–1.02	0.30
Gender												
Male	1.00	–	–	1.00	–	–	1.00	–	–	1.00	–	–
Female	1.42	0.72–2.81	0.31	0.75	0.34–1.68	0.49	0.88	0.41–1.89	0.74	0.97	0.54–1.76	0.92
Highest level of education completed												
None or elementary school	1.00	–	–	1.00	–	–	1.00	–	–	1.00	–	–
Secondary school	2.21	0.95–5.15	0.07	0.46	0.16–1.37	0.16	1.26	0.41–3.89	0.68	0.94	0.42–2.11	0.88
College technical school or university	2.15	0.81–5.75	0.13	0.47	0.14–1.55	0.21	0.93	0.26–3.28	0.91	1.74	0.69–4.37	0.24
Current work												
Employed and/or self-employed	1.00	–	–	1.00	–	–	1.00	–	–	1.00	–	–
Unemployed, social security, and/or invalidity	0.30	0.08–1.11	0.07	0.73	0.13–3.91	0.71	1.72	0.31–9.52	0.53	0.49	0.13–1.82	0.29
Retired and/or stays home by choice	0.78	0.33–1.81	0.56	0.79	0.29–2.13	0.64	1.09	0.43–2.74	0.86	1.04	0.50–2.17	0.91
Annual gross family income												
< $20,000	1.00	–	–	1.00	–	–	1.00	–	–	1.00	–	–
$20,000–$50,000	0.67	0.25–1.76	0.41	0.81	0.27–2.41	0.70	1.17	0.39–3.48	0.78	0.85	0.38–1.93	0.70
> $50,000	0.51	0.16–1.62	0.25	1.47	0.42–5.10	0.55	0.89	0.26–3.05	0.85	0.64	0.24–1.71	0.37
Clinical characteristics												
Intervention program												
Passive diffusion	1.00	–	–	1.00	–	–	1.00	–	–	1.00	–	–
Facilitation	0.40	0.12–1.34	0.14	1.71	0.51–5.65	0.38	0.47	0.15–1.47	0.19	1.10	0.44–2.74	0.85
CVD risk category												
Moderate	1.00	–	–	1.00	–	–	1.00	–	–	1.00	–	–
High	1.20	0.40–3.63	0.75	0.26	0.07–1.01	0.05	0.39	0.12–1.25	0.11	0.85	0.34–2.16	0.73
Diabetes	0.76	0.25–2.28	0.62	0.99	0.19–5.13	0.99	1.19	0.37–3.84	0.77	1.20	0.45–3.20	0.72
Uncontrolled diabetes^1^	1.12	0.44–2.89	0.81	2.89	0.69–12.07	0.15	1.31	0.46–3.75	0.61	1.30	0.53–3.14	0.57
Hypertension	1.20	0.45–3.20	0.72	1.36	0.42–4.40	0.60	3.36	1.12–10.04	0.03	1.14	0.50–2.59	0.76
Uncontrolled hypertension^2^	1.06	0.50–2.23	0.88	1.08	0.47–2.49	0.86	0.39	0.15–1.01	0.05	1.09	0.58–2.06	0.79
Dyslipidemia*	–	–	–	–	–	–	–	–	–	1.47	0.34–6.41	0.61
Uncontrolled dyslipidemia^3^	0.75	0.37–1.52	0.43	0.83	0.37–1.89	0.66	0.53	0.23–1.23	0.14	1.91	0.63–2.26	0.59
Current smokers	0.38	0.17–0.87	0.02	0.62	0.19–2.03	0.42	0.83	0.31–2.25	0.72	0.56	0.24–1.31	0.18
Body mass index												
< 25 kg/m^2^	1.00	–	–	1.00	–	–	1.00	–	–	1.00	–	–
25–30 kg/m^2^	1.06	0.39–2.91	0.91	4.79	0.82–23.87	0.08	0.95	0.30–3.01	0.93	0.65	0.23–1.82	0.41
≥ 30 kg/m^2^	1.39	0.50–3.88	0.53	4.42	0.82–23.87	0.08	1.71	0.56–5.27	0.35	0.70	0.26–1.92	0.48

Abbreviations: CI, confidence interval; CVD, cardiovascular disease; n, number; QoL, quality of life; OR, odds ratio

*388 (97%), 170 (96%), and 270 (96%) patients have dyslipidemia for the indicators “meeting with the nurse to discuss lifestyle habits”, “impact of chronic health conditions on the patient’s quality of life”, and “personal lifestyle change target(s)”, respectively

^1^Fasting blood glucose > 7.0 mmol/L, glycated hemoglobin > 7% and/or glucose 2 h post-prandial > 10.0 mmol/L

^2^Blood pressure ≥ 140/90 mmHg or ≥ 130/80 mmHg if diabetes and/or kidney disease

^3^Low-density lipoprotein cholesterol ≥2 mmol/L and/or apolipoprotein B ≥ 0.8gl/L

## Discussion

To our knowledge, our study is the first to assess the concordance of care processes evaluated using medical records and patient SAQs in the context of an intervention program aimed at improving quality of care. Quality of care, as measured by the TRANSIT indicators, varied substantially between medical records and patient SAQ. Concordance between the two data sources was poor for the subscale and overall compliance scale scores. Seven out of ten pairs of indicators had lower individual compliance scores when evaluated through medical record review. Three pairs of indicators related to care processes delivered exclusively by the nurse had higher individual compliance scores when assessed using medical records. Except for two clinical characteristics, (i.e. smoking status and diagnosis of hypertension), GLMM showed that, among patients who received care based on the medical record, agreement between the two data sources were not affected by patients’ sociodemographic and clinical characteristics.

Agreement varied substantially between the ten pairs of indicators and was poor for subscales and overall compliance scale scores. These findings are supported by another study evaluating the concordance between medical records and patient self-reports for diagnoses, clinical services delivered, counselling and referrals as well as medication use [14]. As in our study, authors found substantial variations both across and within domains of medical care. However, compared to the overall compliance scale score obtained in our study (ICC [95% CI]: 0.46 [0.40–0.53]), Tisnado and al. concluded that the total concordance between the two data sources was fair to good (kappa = 0.5 [0.5–0.6] and total agreement = 80%). This could be explained by the coefficients used to evaluate concordance (ICC versus kappa) and the domains being assessed in each study.

Compared to the indicators evaluated using patient SAQs, most indicators evaluated through medical record review had lower individual compliance scores, suggesting that clinicians may not consistently record care processes in the medical record. When carefully examining the ten pairs of TRANSIT indicators used in this study, we noticed they were all related to counseling and referrals to some extent. Other studies have reported that these interventions are poorly documented in medical records compared to patient surveys. An observational study including 4454 patients treated by 138 physicians showed that the sensitivity of medical records was low for measuring health habit counseling and moderate for physical examination, laboratory testing, and immunization [2]. Another study conducted in an academic family practice clinic also concluded that the most common physician-patient disagreements concerned counseling or treatment procedure [26]. Finally, a study including 1270 patients sampled from 39 medical organizations found that, at the domain-level, counseling and referrals had the worst concordance [14].

Three indicators in the category “motivational interviewing and support for healthy lifestyle change” had higher individual compliance scores when they were documented using medical records. These three indicators (IND79, IND82, and IND89) evaluated the compliance of care processes that were strictly provided by nurses. These results suggest that nurses may be more rigorous when documenting their interventions in the medical record. This finding is supported by a study aimed at evaluating the documentation of patient pain in the emergency department [27]. Indeed, in this study, nurses were 2.2 times more likely to document pain assessment after therapy than physicians (30% versus 16% p < 0.001) [27]. Another study comparing nurses’ and physicians’ documentation of functional abilities of older patient in acute care showed that nurses took more responsibilities in the documentation of the impairment of both Activities of Daily Living and Instrumental Activities of Daily Living [28].

In our study, sociodemographic characteristics were not associated with agreement between medical records and patient SAQ. Some studies found comparable results [9] while others found differences in the odds of agreement by patient demographics such as age, sex, and level of education [11, 12, 29–32]. In terms of clinical characteristics, smoking was associated with reduced agreement between medical records and patient SAQs while a diagnosis of hypertension was associated with increased odds of concordance between the two data sources. Patient motivation, which is influenced by a patient’s physical and psychological state, has been found to affect reporting [33]. In our study, hypertensive patients might have been motivated to change their lifestyle habits. This might explain why they were more likely to accurately report a personal target for lifestyle change**.** Alternatively, sensitive topics such as weight have been found to affect agreement between patients’ surveys and medical records [14, 34]. In our study, *s*mokers might have omitted to report meeting with the nurse to discuss lifestyle habits to avoid exposing their non-adherence to recommendations related to smoking cessation.

### Strengths and limitations

This study was conducted as part of an intervention program aimed at improving CVD prevention in primary care. It focuses on care processes that consider priorities for action and challenges such as the implementation of collaborative practices and the provision of appropriate support for lifestyle changes [16, 17]. The reliability of the indicators documented through medical record review were previously assessed in a psychometric analysis [20, 21]. Despite these strengths, our study is subject to potential limitations. Only ten indicators in the complete set of TRANSIT indicators were evaluated through both medical record review and patient SAQs. Missing data in patient SAQs (i.e. up to 33% for IND29) reduced the number of patients included in our analyses. Since neither data source could be considered as a gold standard, it was impossible to determine whether it was the patient (overreporting) or the clinician (underrecording) who was responsible for the discrepancy between the patient SAQ and the medical record. Thus, the way in which indicators are rated (yes/no/not applicable) made it impossible to know if care processes did not comply with the indicators or if the information needed to evaluate the compliance was not found in the medical record during the study period. While this study was conducted in a complex population of multimorbid patients at moderate or high risk of CVD with at least one uncontrolled CVD risk factor, the findings might not be generalizable to the population treated in primary care.

## Conclusions

To our knowledge, this is the first study evaluating the concordance of care processes based on medical records and patient SAQs in the context of an intervention program aimed at improving CDV prevention in primary care. Our results suggest that medical record and patient SAQ overall agreement is poor. Therefore, when developing and evaluating the effectiveness of quality improvement strategies, researchers must acknowledge that care processes may not be consistently recorded in medical records. They must also be aware that the evaluation of the quality of care may vary depending on the source of information, the clinician responsible of documenting the interventions, and the domain of care (e.g. treatment, diagnosis, management, medication, counseling, referrals, etc.). Our results also suggest that patients’ sociodemographic and clinical characteristics do not affect the reporting of care processes.

## Additional files

Additional file 1: Supplemental Methods. (DOCX 27 kb)

Additional file 2: **Table S1.** Complete list of TRANSIT indicators and their data sources. (DOCX 25 kb)

## Abbreviations

95% CI: 95% confidence interval
BMI: Body mass index
CSSSL: *Centre de santé et de services sociaux de Laval*
CVD: Cardiovascular disease
EMR: Electronic medical record
FMG: Family medicine group
GLMM: Generalized linear mixed model
ICC: Intraclass correlation coefficient
IFT: Interprofessional facilitation team
IND: Indicator
OR: Odd ratio
PABAK: Prevalence-adjusted bias-adjusted kappa
SAQ: Self-administered questionnaire
t_14_: End of the study
