# Supplementary material for: Concordance of care processes between medical records and patient self-administered questionnaires
Source: BMC Fam Pract. 2019 Jul 3;20:92. doi: 10.1186/s12875-019-0979-7 (PMC6607524; doi:10.1186/s12875-019-0979-7)
Supplement: Supplementary file 1 — Supplemental Methods. (DOCX 27 kb) [file 12875_2019_979_MOESM1_ESM.docx]

**SUPPLEMENTAL METHODS**

An initial list of indicators was developed by researchers based on a literature review (1-3) and Canadian cardiovascular treatment guidelines (4-15). Then, to assess the relevance of each indicator, researchers (n = 7), health care managers (n = 6), primary care clinicians (n = 15), patients (n = 2), and family members (n = 2) participated in a two-round RAND/UCLA appropriateness survey (16). The research team sampled experts in the primary care community of Laval, third largest city in the province of Quebec in Canada, to elicit a diverse range of expertise and experience. Patients and family members were referred by participating clinicians. In the first round, all of the 82 indicators presented to the primary care actors were considered relevant. Seven indicators were then added to monitor implementation of the TRANSIT program. In the second round, all of the 89 indicators presented were deemed appropriate. After the survey, the research team merged two indicators into one, rejected nine indicators because of feasibility issues and added two indicators to consider the importance of meeting with the interprofessional family medicine group (FMG) nurse and pharmacist.

The process produced a set of 81 process indicators grouped into seven domains of CVD prevention: 1) general record keeping (n = 13 indicators); 2) targets and recommendations (n = 12); 3) hypertension management (n = 10); 4) dyslipidemia management (n = 5); 5) diabetes management (n = 16); 6) interprofessional collaboration (n = 16); and 7) motivational interviewing and support to healthy lifestyle change (n = 11). Two indicators in the category “general record keeping” also appear in the categories “hypertension management” and “diabetes management”. TRANSIT indicators are documented using medical records (n = 60), pharmacy renewal charts (n = 4) and self-administered patient questionnaires (n = 17).

Prior to assessing the indicators, five research assistants attended a two week-training session to become familiar with the study protocol, the user’s guide and the TRANSIT indicators evaluation grid. During the training, research assistants reviewed the medical records of four patients per FMG and assessed each indicator. Divergences between research assistants were addressed in a group discussion. Once the training session was completed, the research coordinator spent half a day per week with each research assistant for a period of five weeks. In those sessions, the coordinator randomly selected two patients evaluated by the research assistant in the previous week and independently assessed the indicators. Inter-rater discrepancies were discussed.

**REFERENCES**

1. Ornstein S, Jenkins RG, Nietert PJ, Feifer C, Roylance LF, Nemeth L, et al. A multimethod quality improvement intervention to improve preventive cardiovascular care: a cluster randomized trial. Annals of internal medicine. 2004;141(7):523-32.

2. Burge FI, Bower K, Putnam W, Cox JL. Quality indicators for cardiovascular primary care. The Canadian journal of cardiology. 2007;23(5):383-8.

3. Campbell SM, Ludt S, Van Lieshout J, Boffin N, Wensing M, Petek D, et al. Quality indicators for the prevention and management of cardiovascular disease in primary care in nine European countries. European journal of cardiovascular prevention and rehabilitation : official journal of the European Society of Cardiology, Working Groups on Epidemiology & Prevention and Cardiac Rehabilitation and Exercise Physiology. 2008;15(5):509-15.

4. Culleton B, Drouin D, Larochelle P, Leiter LA, McFarlane P, Tobe S. Canadian Diabetes Association Clinical Practice Guidelines Expert Committee. Treatment of Hypertension. Canadian Diabetes Association 2008 clinical practice guidelines for the prevention and management of diabetes in Canada. Can J Diabetes. 2008;32(Supplement 1):S115-S8.

5. Hackam DG, Khan NA, Hemmelgarn BR, Rabkin SW, Touyz RM, Campbell NR, et al. The 2010 Canadian Hypertension Education Program recommendations for the management of hypertension: part 2 - therapy. The Canadian journal of cardiology. 2010;26(5):249-58.

6. Quinn RR, Hemmelgarn BR, Padwal RS, Myers MG, Cloutier L, Bolli P, et al. The 2010 Canadian Hypertension Education Program recommendations for the management of hypertension: part I - blood pressure measurement, diagnosis and assessment of risk. The Canadian journal of cardiology. 2010;26(5):241-8.

7. Rabi DM, Daskalopoulou SS, Padwal RS, Khan NA, Grover SA, Hackam DG, et al. The 2011 Canadian Hypertension Education Program recommendations for the management of hypertension: blood pressure measurement, diagnosis, assessment of risk, and therapy. The Canadian journal of cardiology. 2011;27(4):415-33 e1-2.

8. Genest J, Frohlich J, Fodor G, McPherson R, Working Group on H, Other D. Recommendations for the management of dyslipidemia and the prevention of cardiovascular disease: summary of the 2003 update. CMAJ : Canadian Medical Association journal = journal de l'Association medicale canadienne. 2003;169(9):921-4.

9. Bowering K, Ekoé J-M, Kalla TP. Canadian Diabetes Association Clinical Practice Guidelines Expert Committee. Foot care. Canadian Diabetes Association 2008 Clinical Practice Guidelines. Can J Diabetes. 2008;32(Supplement 1):S143-S6.

10. Brez S, Berard L, Blumer I. Canadian Diabetes Association Clinical Practice Guidelines Expert Committee. Monitoring glycemic control. Canadian Diabetes Association 2008 Clinical Practice Guidelines. Can J Diabetes. 2008;32(Supplement 1):S32-S6.

11. Canadian Diabetes Association Clinical Practice Guidelines Expert Committee. Canadian Diabetes Association 2008 clinical practice guidelines for the prevention and management of diabetes in Canada. Can J Diabetes. 2008;32(Supplement 1):S1-S201.

12. Feig DS, Palda VA, Lipscombe L, Canadian Task Force on Preventive Health C. Screening for type 2 diabetes mellitus to prevent vascular complications: updated recommendations from the Canadian Task Force on Preventive Health Care. CMAJ : Canadian Medical Association journal = journal de l'Association medicale canadienne. 2005;172(2):177-80.

13. Imran S, Ross S. Canadian Diabetes Association Clinical Practice Guidelines Expert Committee. Targets for glycemic control. Canadian Diabetes Association 2008 Clinical Practice Guidelines. Can J Diabetes. 2008;32(Supplement 1):S29-S31.

14. Jones H, Berard L, Nichol H. Canadian Diabetes Association Clinical Practice Guidelines Expert Committee. Self-management education. Canadian Diabetes Association 2008 Clinical Practice Guidelines. Can J Diabetes. 2008;32(Supplement 1):S25-S8.

15. Ur E, Chiasson J, Ransom T, Rowe R. Canadian Diabetes Association Clinical Practice Guidelines Expert Committee. Screening for Type 1 and Type 2 Diabetes. Canadian Diabetes Association 2008 clinical practice guidelines for the prevention and management of diabetes in Canada. Can J Diabetes. 2008;32(Supplement 1):S14-S6.

16. Fitch K, Bernstein SJ, Aguilar MD, Burnand B, LaCalle JR, Lazaro P, et al. The RAND/UCLA appropriateness method user's manual. Santa Monica,California,United States: RAND Corp.; 2001.
