# Supplementary material for: Concordance of care processes between medical records and patient self-administered questionnaires
Source: BMC Fam Pract. 2019 Jul 3;20:92. doi: 10.1186/s12875-019-0979-7 (PMC6607524; doi:10.1186/s12875-019-0979-7)
Supplement: Supplementary file 2 — Table S1. Complete list of TRANSIT indicators and their data sources. (DOCX 25 kb) [file 12875_2019_979_MOESM2_ESM.docx]

**Table S1.** Complete list of TRANSIT indicators and their data sources

| **Codes** | **Indicators** | **Data sources** | | |
| --- | --- | --- | --- | --- |
|  |  | **MR** | **PR** | **SAQ** |
| **General record keeping** | | | | |
| IND01 | Height | ✓ |  |  |
| IND02 | Weight | ✓ |  |  |
| IND03 | Body mass index | ✓ |  |  |
| IND06 | Waist circumference | ✓ |  |  |
| IND09 | Smoking status | ✓ |  |  |
| IND12 | Alcohol consumption | ✓ |  |  |
| IND15 | Salty foods or salt consumption | ✓ |  |  |
| IND17 | Foods rich in fat and cholesterol consumption | ✓ |  |  |
| IND19 | Physical activity intensity level | ✓ |  |  |
| IND22 | Stress intensity level | ✓ |  |  |
| IND24 | Estimated cardiovascular disease risk^1^ | ✓ |  |  |
| **Targets and recommendations** | | | | |
| IND04 | Weight and/or body mass index target(s) | ✓ |  |  |
| IND07 | Waist circumference target(s) | ✓ |  |  |
| IND08 | Recommendation for lifestyle changes to reduce weight and/or waist circumference | ✓ |  |  |
| IND10 | Smoking cessation target(s) | ✓ |  |  |
| IND11 | Recommendations for lifestyle changes to stop smoking | ✓ |  |  |
| IND13 | Alcohol consumption target(s) | ✓ |  |  |
| IND14 | Recommendation for lifestyle changes to reduce alcohol consumption | ✓ |  |  |
| IND16 | Recommendation for lifestyle changes to reduce sodium intake | ✓ |  |  |
| IND18 | Recommendation for lifestyle changes to reduce fat and cholesterol intake | ✓ |  |  |
| IND20 | Physical activity target(s) | ✓ |  |  |
| IND21 | Recommendation for lifestyle changes to increase physical activity | ✓ |  |  |
| IND23 | Recommendation for lifestyle changes to reduce stress | ✓ |  |  |
| **Hypertension management** | | | | |
| IND26 | BP measurements as recommended in guidelines^2^ | ✓ |  |  |
| IND28 | BP target(s) | ✓ |  |  |
| IND29 | Instructions for home BP monitoring received |  |  | ✓ |
| IND30 | Instructions for home BP monitoring | ✓ |  |  |
| IND31 | Recommendation for BP diary | ✓ |  |  |
| IND32 | BP diary completed |  |  | ✓ |
| IND34 | Change in BP treatment plan^3^ if BP >140/90 or >130/80 and diabetes or kidney disease | ✓ |  |  |
| IND35 | Change in BP medication by a clinician if BP >140/90 or >130/80 and diabetes or kidney disease | ✓ |  |  |
| IND36 | Pharmaceutical opinion on BP medication if the adherence to pharmacological treatment is sub-optimal (<80% or >120%) |  | ✓ |  |
| IND37 | Pharmaceutical opinion on BP medication | ✓ |  |  |
| **Dyslipidemia management** | | | | |
| IND39 | LDL-C target(s) | ✓ |  |  |
| IND42 | Change in lipid-lowering treatment plan^3^ if LDL-C ≥2 mmol/L or apo B ≥0.8 g/L | ✓ |  |  |
| IND43 | Change in lipid-lowering medication by a clinician if LDL-C ≥2 mmol/L or apo B ≥0.8 g/L | ✓ |  |  |
| IND44 | Pharmaceutical opinion on lipid-lowering medication if the adherence to pharmacological treatment is sub-optimal (<80% or >120%) |  | ✓ |  |
| IND45 | Pharmaceutical opinion on lipid lowering treatment | ✓ |  |  |
| **Diabetes management** | | | | |
| IND46 | Blood glucose or HbA1c measurements as recommended in the guidelines^4^ | ✓ |  |  |
| IND48 | HbA1c or blood glucose target(s) | ✓ |  |  |
| IND49 | Instructions for home blood glucose monitoring received |  |  | ✓ |
| IND50 | Instructions for home blood glucose monitoring | ✓ |  |  |
| IND51 | Recommendation for blood glucose diary | ✓ |  |  |
| IND52 | Blood glucose diary completed |  |  | ✓ |
| IND54 | Change in diabetes treatment plan^3^ if HbA1c >7% or fasting plasma glucose >7 mmol/L | ✓ |  |  |
| IND55 | Change in diabetes medication by a clinician if HbA1c >7% or fasting plasma glucose >7 mmol/L | ✓ |  |  |
| IND56 | Pharmaceutical opinion on diabetes medication if the adherence to pharmacological treatment is sub-optimal (<80% or >120%) |  | ✓ |  |
| IND57 | Pharmaceutical opinion on diabetes treatment | ✓ |  |  |
| IND58 | Education on self-management for diabetes received |  |  | ✓ |
| IND59 | Education on self-management for diabetes | ✓ |  |  |
| IND60 | Foot exam completed by a nurse |  |  | ✓ |
| IND61 | Recommendation for a foot exam by a nurse | ✓ |  |  |
| IND62 | Retina examination in the past 2 years |  |  | ✓ |
| IND63 | Recommendation for a retina examination in the past 2 years | ✓ |  |  |
| **Interprofessional collaboration** | | | | |
| IND64 | Therapeutic nursing plan | ✓ |  |  |
| IND65 | Nurse follow-up with the patient’s family | ✓ |  |  |
| IND66 | Referral to a clinician other than a physician and a nurse | ✓ |  |  |
| IND67 | Referral to a clinician other than a physician and a nurse |  |  | ✓ |
| IND68 | Meeting with a clinician other than a physician and a nurse |  |  | ✓ |
| IND69 | Note from a clinician other than a physician and a nurse | ✓ |  |  |
| IND70 | Referral to a FMG workshop and/or a group class offered by the local care center^5^ | ✓ |  |  |
| IND71 | Referral to a group class offered by the local care center^5^ |  |  | ✓ |
| IND72 | Participation to a group class^5^ offered by the local care center |  |  | ✓ |
| IND73 | Referral to a community resource^6^ | ✓ |  |  |
| IND74 | Referral to a community resource^6^ |  |  | ✓ |
| IND75 | Community resource used by the patient |  |  | ✓ |
| IND76 | Laboratory tests ordered by FMG nurse according to collective prescription | ✓ |  |  |
| IND77 | Dose adjustment by pharmacist according to collective prescription |  | ✓ |  |
| IND90 | Consultation with FMG nurse | ✓ |  |  |
| IND91 | Consultation with FMG pharmacist | ✓ |  |  |
| **Motivational interviewing and support for healthy lifestyle change** | | | | |
| IND79 | Meeting with a nurse to discuss lifestyle habits | ✓ |  |  |
| IND80 | Meeting with a nurse to discuss lifestyle habits |  |  | ✓ |
| IND81 | Motivational interviewing technique applied by nurse | ✓ |  |  |
| IND82 | Impact of chronic health conditions^7^ on the patient’s quality of life | ✓ |  |  |
| IND83 | Impact of chronic health conditions^7^ on the patient’s quality of life |  |  | ✓ |
| IND84 | Personal lifestyle changes target(s) | ✓ |  |  |
| IND85 | Nurse follow-up to discuss lifestyle changes | ✓ |  |  |
| IND86 | Personal lifestyle changes target(s) |  |  | ✓ |
| IND87 | Patient’s adherence to targeted lifestyle changes | ✓ |  |  |
| IND88 | TRANSIT health booklet^8^ used |  |  | ✓ |
| IND89 | TRANSIT health booklet^8^ used | ✓ |  |  |

Abbreviations: apo B, apolipoprotein B; BP, blood pressure; FMG, family medicine group; HbA1c, glycated hemoglobin; LDL-C, low-density lipoprotein cholesterol; MR, medical record; PR, pharmaceutical record; SAQ, self-administered questionnaire.

^1^Assessed by the Framingham score

^2^Once a year for non-hypertensive patients; every 3 to 6 months for hypertensive patients with non-pharmacological treatment; every 3 to 6 months for well controlled hypertensive patients; and once a month or every 2 months until 2 consecutive normal readings for uncontrolled hypertensive patients

^3^Medication or lifestyle changes and/or re-assessment plan

^4^Fasting plasma glucose measured once in the past 3 years for non-diabetic patient; glucose tolerance test if fasting blood glucose between 6.1-6.9 with suspected type II diabetes or glucose intolerance; HbA1c every 3 months for patient with uncontrolled diabetes; and HbA1c every 6 months for patient with controlled diabetes

^5^Group classes offered by the local care center include group class on diabetes, dyslipidemia, diabetes, healthy weight management, smoking habits

^6^Community resources include resources for nutrition (organization) and physical activity (recreational center and walking club) as well as phone line for diabetes, depression, physiological help, physical activity, smoking cessation, and nutrition

^7^Chronic disease or risk factor

^8^The TRANSIT health booklet is a personalized tool that allows the patient receiving care from multiple clinicians in various locations to share his/her medical information. It also helps the patient keep track of his/her progress.
